# Prevention of glucocorticoid-associated osteonecrosis by intravenous administration of mesenchymal stem cells in a rabbit model

**DOI:** 10.1186/s12891-017-1837-1

**Published:** 2017-11-21

**Authors:** Shusuke Ueda, Miyako Shimasaki, Toru Ichiseki, Yoshimichi Ueda, Masanobu Tsuchiya, Ayumi Kaneuji, Norio Kawahara

**Affiliations:** 1 Department of Orthopaedic Surgery, Kanazawa Medical University, Daigaku 1-1, Uchinada-machi, Kahoku-gun, Ishikawa 920-0293 Japan; 2 Department of Phathology 2, Kanazawa Medical University, Daigaku 1-1, Uchinada-machi, Kahoku-gun, Ishikawa 920-0293 Japan

**Keywords:** Osteonecrosis, Glucocorticoid, Mesenchymal stem cell (MSC), Green fluorescent protein (GFP), Homing, Intravenous administration

## Abstract

**Background:**

Glucocorticoid-associated osteonecrosis is an intractable condition, making the establishment of preventative strategies of particular importance. Recently various studies using mesenchymal stem cells (MSC) have been conducted. Using a rabbit glucocorticoid-associated osteonecrosis model we administered green fluorescent protein (GFP)-labeled MSC intravenously to investigate their effect on osteonecrosis.

**Methods:**

A rabbit osteonecrosis model in which methylprednisolone (MP) 20 mg/kg was injected into the gluteus of a Japanese white rabbit was used. Simultaneously with MP, MSC labeled with GFP (GFP-labeled MSC) were injected intravenously. Fourteen days later the animals were killed (MSC(+)/MP(+)/14d), femurs were extracted, and the prevalence of osteonecrosis was determined histopathologically. Also, animals were killed 3 days after simultaneous administration of GFP-labeled MSC and MP (MSC(+)/MP(+)/3d), and western blotting (WB) for GFP was performed of the femur, liver, kidney, lung, blood vessel, and vertebra, in addition to immunohistochemical study of femur. As a control for the histopathological study, animals were killed 14 days after MP administration and intravenous vehicle injection (MSC(−)/MP(+)/14d). For WB, animals were killed 3 days after intravenous GFP-labeled MSC administration and vehicle injection into the gluteus (MSC(+)/MP(−)/3d).

**Results:**

In MSC(−)/MP(+)/14d osteonecrosis was found in 7 of 10 rabbits (70%), while in MSC(+)/MP(+)/14d, partial bone marrow necrosis was found in only 1 rabbit (12.5%); osteonecrosis was not found in 7 of 8 rabbits (*p* < 0.05). WB showed expression of GFP in the femur, not in the liver, kidney, lung, blood vessel, or vertebra, of MSC(+)/MP(+)/3d; expression of GFP-labeled MSC was absent in the femur of MSC(+)/MP(−)/3d. In the immunohistochemical study of MSC(+)/MP(+)/3d, homing of GFP-labeled MSC was noted perivascularly in the femur, but not in MSC(+)/MP(−)/3d.

**Conclusions:**

With transvenous MSC administration a significant prophylactic effect against glucocorticoid-associated osteonecrosis was found. Direct administration of MSC to the site of tissue injury requires highly invasive surgery. In contrast, as shown here the simple and hardly invasive intravenous administration of MSC may succeed in preventing osteonecrosis.

**Electronic supplementary material:**

The online version of this article (10.1186/s12891-017-1837-1) contains supplementary material, which is available to authorized users.

## Background

A glucocorticoid-associated femoral head necrosis leads to destruction of the femoral head. Once it develops and the femoral head is collapsed, surgery, such as osteotomy or artificial joint placement, becomes the sole option to treat the associated pain. This highlights the importance of the prevention, though at present no effective strategies to prevent osteonecrosis in patients who require therapeutic steroids have yet been established.

Glucocorticoid-associated femoral head necrosis is generally attributed to ischemia, with the underlying mechanisms proposed to include oxidative stress, abnormalities of lipid metabolism, and vascular injury [1–3]. Diverse studies have been undertaken focusing not only on elucidation of its pathophysiology but also preventative and therapeutic strategies. With regard to prevention the administrations of vitamin E, pitavastatin, and other agents have been attempted [4, 5]. Although with agents such as vitamin E and pitavastatin an osteonecrosis inhibitory effect is obtained (about 34% inhibition with vitamin E, about 53% with statin administration), sufficient inhibition has not yet been achieved [6, 7].

Recently in various fields the systemic administration of mesenchymal stem cells (MSC) or their local administration by surgical implantation has been studied for both therapeutic and prophylactic purposes. MSC are somatic stem cells distributed throughout the body. They are characterized by multipotency allowing them to differentiate into various cell types including osteoblasts and osteocytes, as well as marked self-duplicating ability, and replication competence [8]. These properties make it possible to use autologous stem cells of the patient himself to avoid immune reactions. Furthermore, since these cells migrate to sites of injury or inflammation [9–15], development of an excessive immunoreaction is inhibited [10, 14], and they are believed to play a role in the repair of injured and ischemic tissues [9, 15, 16], and also have been reported to produce various growth factors and cytokines that contribute to tissue healing [11, 15–17]. However, actual clinical application of stem cell implantation would require surgery, which is associated with high invasiveness. Considering the characteristic homing of MSC at injured sites, their delivery to such sites where osteonecrosis develops by systemic administration via the transvenous route appears to be a reasonable strategy. Also, transvenous administration would preclude surgical intervention, making the procedure extremely low-invasive and convenient.

These considerations prompted us to investigate the distribution of MSC in individual organs by intravenously administering MSC in a glucocorticoid-associated rabbit osteonecrosis model and to prevent the development of osteonecrosis.

## Methods

### Animals

The experimental protocol for this study was approved by the Animal Research Committee of Kanazawa Medical University (#2016-75). A commonly used Japanese white rabbit (Sankyo Labo Service, Tokyo, Japan) osteonecrosis model (mean rabbit weight of 3.5 kg) in which a single dose of methylprednisolone (MP) 20 mg/kg was injected into the gluteus was used [2]. Japanese white rabbits to which MSC had been administered to an auricular vein and MP to the gluteus were killed 14 days after administration (MSC(+)/MP(+)/14d), and femurs of 8 rabbits were extracted. As a control, a rabbit osteonecrosis model in which vehicle (physiological saline) was injected into an auricular vein and the animals killed 14 days after administration (MSC(−)/MP(+)/14d) (10 rabbits) was used. Also, animals were killed 14 days after intravenous vehicle injection to the gluteus (MSC(−)/MP(−)/14d). In WB and immunohistochemical study, animals were killed 3 days after administration of green fluorescent protein (GFP)-labeled MSC and MP (MSC(+)/MP(+)/3d), and administration of GFP-labeled MSC and vehicle (MSC(+)/MP(−)/3d). All rabbits were killed using an overdose of intravenously injected sodium pentobarbital.

### Cell culture and GFP-labelling of mesenchymal stem cells

Rabbit mesenchymal stem cells (Cyagen, DS Pharma Biomedical, Tokyo, Japan) were maintained as a subconfluent monolayer culture in mesenchymal stem cell growth medium (Cyagen, DS Pharma Biomedical, Tokyo, Japan) at 37 °C under 5% CO_2_ / 95% air. The medium was exchanged every 3 days, and passaging was routinely performed when the culture reached 70% confluency. The rabbit mesenchymal stem cells were made to express GFP by using pJTI™ R4 Exp CMV EmGFP pA Vector (Invitrogen by Life Technology, Tokyo, Japan) and Effectene Transfection Reagent (QIAGEN, Tokyo, Japan). An additional figure file shows this in more detail (see Additional file 1). After the culture reached 80% confluency, the rabbit was injected with GFP-labeled rabbit mesenchymal stem cells with 1.7 × 10^7^ in a total volume of 1 ml Mesenchymal Stem Cell Growth medium into an auricular vein. At the same time, the rabbit was injected into the gluteal muscle with MP. After 3 days, specimens were obtained from the femur, vertebra, kidney, liver, blood vessel, and lung. They were formalin-fixed and paraffin-embedded for immunostaining, or frozen for western blotting (MSC(+)/MP(+)/3d) (*n* = 3). As a control we used Japanese white rabbits intravenously injected with GFP-labeled MSC and vehicle into the gluteus that were killed 3 days after administration (MSC(+)/MP(−)/3d) (*n* = 3), and then examined in the same way.

### Histopathology

Femurs were extracted 14 days after the simultaneous administration of MSC and MP (MSC(+)/MP(+)/14d). As a control group, femurs were extracted 14 days after administration of MP and intravenous vehicle injection (MSC(−)/MP(+)/14d). Also, femurs were extracted 14 days after intravenous vehicle injection to the gluteus (MSC(−)/MP(−)/14d). On the same day as the animals were killed, the extracted femurs were sliced in planes parallel to the coronal plane of the femoral neck, and tissue fixation was performed by immersing the specimens in 10% buffered formalin for 1 week. Then, after decalcification in formic acid, the specimens were embedded in paraffin and cut into 4-μm-thick sections with a microtome. Necrosis of bone and marrow tissues was examined in hematoxylin and eosin (H&E)-stained preparations light microscopically by the two pathologists without information on the experimental condition. Also, two pathologists made the evaluation using each 3 slides. The definition of osteonecrosis used in this model was based on that of Yamamoto et al. [2]. Namely osteonecrosis was considered to be present when necrosis of medullary haematopoietic cells and fat cells, and/or osteocyte empty lacunae and condensed nuclei were evident.

### Western blotting

Immunoblotting for GFP was performed on the rabbit mesenchymal stem cells which showed expression of GFP and 6 rabbit tissues: the femur, vertebra, kidney, liver, blood vessel and lung. Proteins were extracted using protein extraction solution (PRO-PREP™, iNtRON Biotechnology, Kyungki-Do, Korea). Extracted protein (20 μg) was applied to and electrophoresed on a 10% polyacrylamide gel, and transferred to a nitrocellulose membrane (Atoh, Tokyo, Japan). The membranes were reacted overnight at 4 °C with anti-GFP mouse monoclonal antibody (Abcam 38,689, Tokyo, Japan) at a concentration of 0.5 μg/ml. After incubation with peroxidase-labeled goat anti-mouse IgG antibody (Dako Cytomation, Santa Clara, USA) for 1 h at the room temperature and vigorous washing, the nitrocellulose membrane was incubated with Chemiluminescence Luminol Reagent (Immuno Star LD, Wako, Tokyo, Japan) and photographed digitally using ImageQuant LAS 4000 mini (GE healthcare Japan Co, Tokyo, Japan). All samples were standardized by immunoblot using anti-actin mouse monoclonal antibody (Sigma Chemical Co., St. Louis, MO).

### Immunostaining for GFP

Formalin-fixed rabbit mesenchymal stem cells which had a gene that strongly expressed GFP were used as a positive control of the immunostaining. Immunostains for GFP were performed according to the datasheet of Anti-GFP antibody [6AT316] (Abcam 38,689). For antigen retrieval, tissue sections were heated at 95 °C for 40 min. After washing in PBS, endogenous peroxidase activity was inhibited with normal rabbit serum (Nichirei, Tokyo, Japan) for 10 min. The sections were incubated overnight at 4 °C with affinity-purified anti-GFP mouse monoclonal antibodies (Abcam 38,689) at a concentration of 10 μg/ml. The sections were incubated with 10 μg/ml biotinylated rabbit anti-mouse immunoglobulin (Nichirei) for 30 min. After washing in PBS, they were incubated with 100 μg/ml horseradish peroxidase-conjugated streptavidin (Nichirei Co.) for 30 min. The color reaction was performed with 0.05 Tris-HCl (pH 7.6) containing 3,3′-diaminobendizine tetrahydrochloride (Nichirei) and the sections were counterstained with Meyer’s hematoxylin. A BX53 microscope (Olympus, Tokyo, Japan) and a DP71 camera (Olympus) were used.

### Statistical analysis

Significant differences in the osteonecrosis development rate in the H&E-stained specimens, were determined by Fisher’s exact test. Significance was defined as *p* < 0.05.

## Results

### Inhibition of osteonecrosis by MSC

**Fig. 1:**
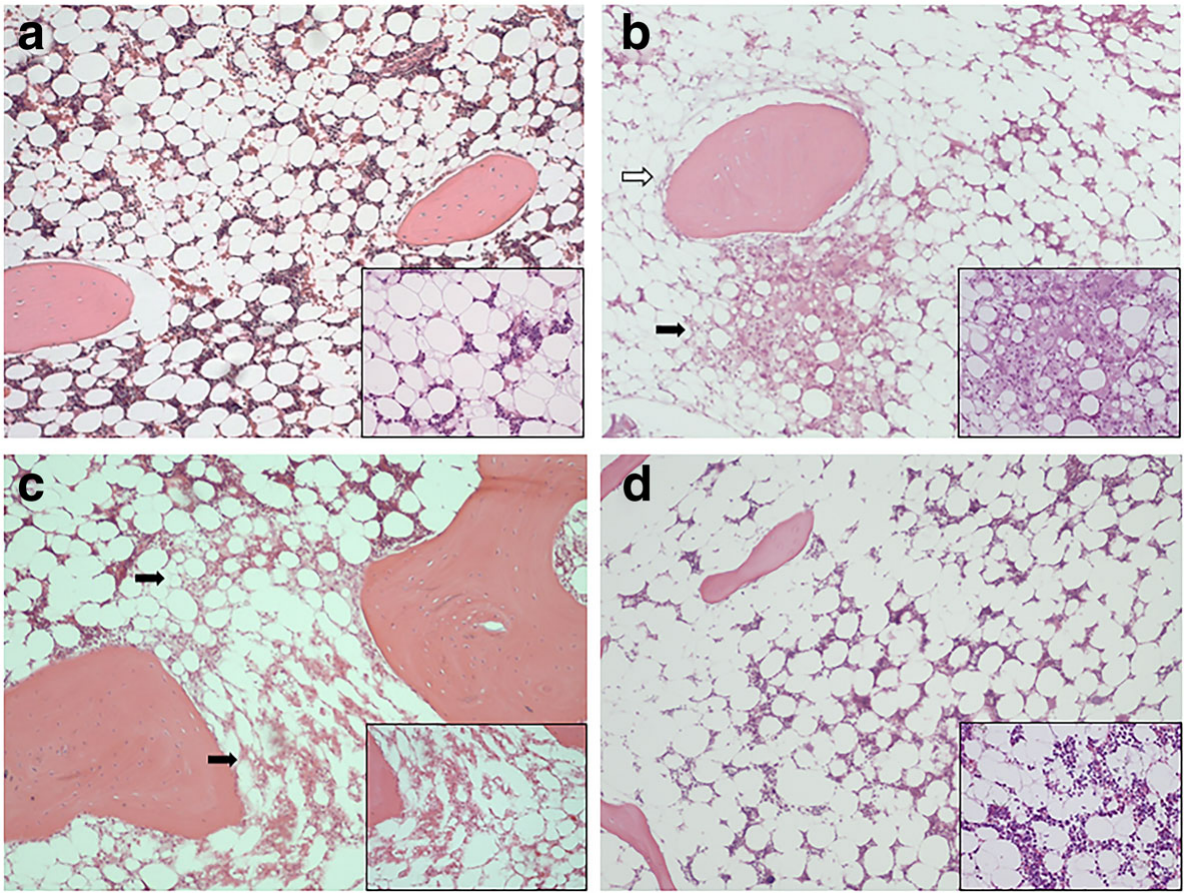
Histopathological analysis of the influence of MSC in a glucocorticoid-associated rabbit osteonecrosis model (hematoxylin-eosin staining). MSC(−)/MP(−)/14d (**a**). MSC(−)/MP(+)/14d (**b**, **c**). MSC(+)/MP(+)/14d (**d**). Osteonecrosis was considered to be present when necrosis of medullary haematopoietic cells and fat cells (black arrow), and/or osteocyte empty lacunae and condensed nuclei (white arrow) were evident. As a control, MSC(−)/MP(−)/14d was showed (**a**). In MSC(−)/MP(+)/14d, in 6 of 10 rabbits achromatic nuclei and/or empty lacunae characteristic of osteonecrosis were found in osteocytes and bone marrow cells (**b**). Also, in 1 of 10 rabbits bone marrow necrosis was found (**c**). Osteonecrosis was found in a total of 7 of 10 rabbits (70%). In MSC(+)/MP(+)/14d, in 7 of 8 rabbits no bone marrow necrosis or osteonecrosis was evident (**d**), while in only a single specimen (12.5%) were necrotic or degenerated cells found in a part of the bone marrow. In MSC(+)/MP(+)/14d as compared to MSC(−)/MP(+)/14d osteonecrosis was significantly decreased. Magnification: ×100, ×200 (enlarged view)

Since in this model an osteonecrosis development rate of 70% 14 days after MP administration has been reported [1, 18–20], the histopathological study was conducted at this time point. In MSC(−)/MP(+)/14d osteonecrosis was found in 7 of 10 rabbits (70%). In 6 of 10 rabbits both osteonecrosis and bone marrow necrosis were found (Fig 1b), while in 1 of 10 rabbits bone marrow necrosis was found (Fig 1c). An additional figure file shows this in more detail (see Additional file 2). In contrast, in MSC(+)/MP(+)/14d bone marrow necrosis was limited to only a single rabbit (12.5%); in 7 of 8 rabbits no obvious bone marrow necrosis or osteonecrosis was found (Fig. 1d) (*p* < 0.05). An additional figure file shows this in more detail (see Additional file 3). As a control, MSC(−)/MP(−)/14d was showed. In MSC(−)/MP(−)/14d was found no obvious bone marrow necrosis or osteonecrosis (Fig 1a).

### Accumulation of GFP-labeled MSC in the injured femur

**Fig. 2:**
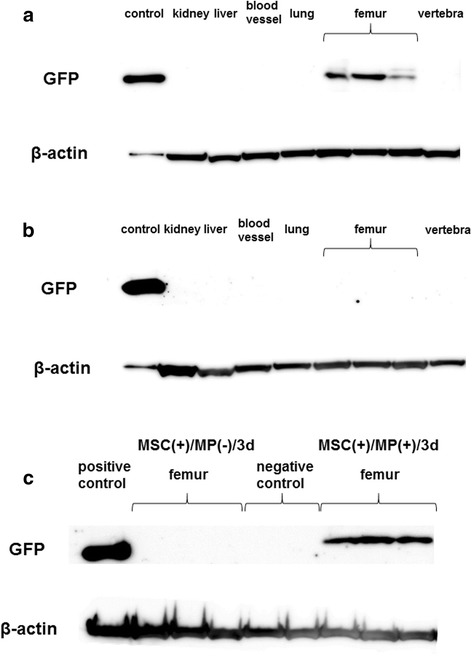
Western blotting of in each organ administered GFP-labeled MSC. Expression of GFP-labeled MSC in each organ (**a**, **b**). MSC(+)/MP(+)/3d (**a**). MSC(+)/MP(−)/3d (**b**). Comparison of MSC(+)/MP(+)/3d and MSC(+)/MP(−)/3d in femur (**c**). In MSC(+)/MP(−)/3d there was no expression of GFP-labeled MSC in femur or any organ, while in MSC(+)/MP(+)/3d GFP-labeled MSC expression was not found in any organ but was significantly present in femur

Previous reports have documented the presence of tissue injury preceding osteonecrosis 3 days after MP administration, with this speculated to be related to the generation of osteonecrosis [1]. For this reason, we chose to investigate the dynamics of MSC by Western blot in MSC(+)/MP(+)/3d. In MSC(+)/MP(+)/3d, no expression of GFP was noted in the liver, kidney, blood vessel, lung, or vertebra, in contrast to which significant expression was found in the femur (Fig. 2a and c). In MSC(+)/MP(−)/3d no expression was found in the femur either (Fig. 2b and c). An additional figure file shows this in more detail (see Additional file 4).

### Homing of GFP-labeled MSC

**Fig. 3:**
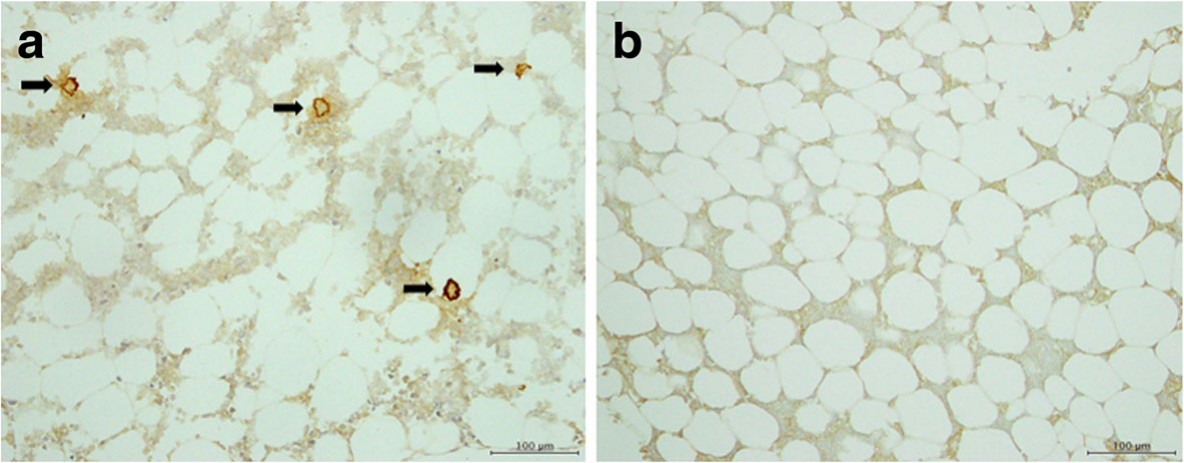
Immunohistochemical study of femur administered GFP-labeled MSC. MSC(+)/MP(+)/3d (**a**). MSC(+)/MP(−)/3d (**b**). GFP-labeled MSC are shown with black arrows. Homing of GFP-labeled MSC was found in the femur with MP administration, the site of tissue injury (**a**). Scale bar: 100 μm

To confirm that homing of MSC occurs at sites of tissue injury, GFP-labeled MSC were subjected to immunohistochemical study (Fig. 3). As shown in Fig. 3, whereas in MSC(+)/MP(−)/3d almost no homing of GFP-labeled MSC was found (Fig. 3b), in MSC(+)/MP(+)/3d significant homing was found in femur (Fig. 3a). An additional figure file shows this in more detail (see Additional file 5). Furthermore homing of MSC was especially prominent in the perivascular structures in the femur.

## Discussion

Various reports have focused on embryonic stem (ES) cell. Regarding the liver, when ES cells are administered via a tail vein in a hepatic injury mouse model, they have been reported to accumulate in the liver, which is the site of injury in this model. ES cells have also been reported to have the ability to promote recovery at the site of injury [21]. In the case of specific injury to the pancreas as well, homing of ES cells to the site of injury, and their exerting a reparative property for injured tissues have been reported [22–24]. MSC also act similarly at sites of injury to the liver [24, 25]. All of these findings support the contention that MSC migrate to sites of injury where they exert a reparative function on injured tissues [26, 27]. Recently, in the orthopedic surgery field as well various multifaceted studies using MSC have been undertaken, focusing on cartilage regeneration, meniscus regeneration, and treatment of spinal injury. In the present experiment an attempt was made to prevent osteonecrosis, which is considered intractable, by the systemic administration of MSC via a transvenous route. At 14 days after the simultaneous administration of MP and MSC the rate of glucocorticoid-associated osteonecrosis was significantly decreased.

To assess the homing of systemically administered MSC at injured sites, in this experiment GFP was inserted by gene transfer into systemically administered MSC, and then the homing of MSC to injury sites confirmed by labeling the GFP. Also, in the co-administration of MP, MSC were demonstrated in the femur, a site of predilection of necrosis both with WB and immunostaining. However, in the absence of MP administration, MSC did not accumulate in any organ, and in contrast to the co-administration of MP, they were not expressed in the liver, kidney, lung, blood vessel, or vertebra. Previous investigations have confirmed that in rabbit osteonecrosis intraosseous injury occurs within 3–5 days after MP administration and is related to necrosis [1]. In the present experiment as well, distribution of MSC to the femur occurred within 3 days, thus making them adequate for the purpose of osteonecrosis prevention.

Since the underlying cause of osteonecrosis is speculated to be ischemia, and since MSC enhance the blood flow to ischemic tissues [28–31], it is reasonable to expect them to exert a considerable prophylactic effect against the development of osteonecrosis. In contrast, direct implantation of MSC at injured sites would be highly invasive and require specialized technique. The transvenous administration used in this study was easily performed and of low invasiveness. Thus if it can be demonstrated to be effective in preventing osteonecrosis, its ready clinical applicability will be a major advantage.

## Conclusions

With transvenous MSC administration a significant prophylactic effect against glucocorticoid-associated osteonecrosis was found. This suggests that by taking advantage of the property of homing at the site of injury, the simple and low-invasive intravenous administration of MSC may succeed in preventing osteonecrosis, it might be also effective for clinical application.

## Additional files

Additional file 1: Figure S1. Character of GFP transfected MSC. Phase contrast image (a, b). Nuclear staining (c). Forced expression of GFP (d). GFP-negative MSC (a, c), GFP-labeled MSC (b, d). GFP was transfected to MSC, and the GFP-labeling was confirmed. After forced expression of GFP, it was confirmed labeling of approximately 60% of MSC (d). (TIFF 445 kb)

Additional file 2: Figure S2. Histopathological analysis in a glucocorticoid-associated rabbit osteonecrosis model. Osteonecrosis was considered to be present when necrosis of medullary haematopoietic cells and fat cells (black arrow), and/or osteocyte empty lacunae and condensed nuclei (white arrow) were evident. In 6 of 10 rabbits achromatic nuclei and/or empty lacunae characteristic of osteonecrosis were found in osteocytes and bone marrow cells (a, b, c, d, e, f). Also, in 1 of 10 rabbits bone marrow necrosis was found (g). In 3 of 10 rabbits no bone marrow necrosis or osteonecrosis was evident (h, i, j). Magnification: ×100. (TIFF 824 kb)

Additional file 3: Figure S3. Histopathological analysis of the influence of MSC in a glucocorticoid-associated rabbit osteonecrosis model. Osteonecrosis was considered to be present when necrosis of medullary haematopoietic cells and fat cells (black arrow), and/or osteocyte empty lacunae and condensed nuclei were evident. In 7 of 8 rabbits no bone marrow necrosis or osteonecrosis was evident (a, b, c, d, e, f, g), while in only a single specimen (12.5%) were necrotic or degenerated cells found in a part of the bone marrow (h). Magnification: ×100. (TIFF 650 kb)

Additional file 4: Figure S4. Western blotting of in each organ administered GFP-labeled MSC. MSC(+)/MP(+)/3d (a, b, c). MSC(+)/MP(−)/3d (d, e, f). In MSC(+)/MP(−)/3d there was no expression of GFP-labeled MSC in femur or any organ, while in MSC(+)/MP(+)/3d GFP-labeled MSC expression was not found in any organ but was significantly present in femur. (TIFF 159 kb)

Additional file 5: Figure S5. Immunohistochemical study of GFP-labeled MSC in femur. MSC(+)/MP(+)/3d (a, b, c). MSC(+)/MP(−)/3d (d, e, f). GFP-labeled MSC are shown with black arrows. Homing of GFP-labeled MSC was found in the femur with MP administration, the site of tissue injury. (TIFF 677 kb)

## Abbreviations

ES: Embryonic stem
GFP: Green fluorescent protein
MP: Methylprednisolone
MSC: Mesenchymal stem cell
WB: Western blotting
