# Supplementary figures and images for: Prevention of glucocorticoid-associated osteonecrosis by intravenous administration of mesenchymal stem cells in a rabbit model
Source: BMC Musculoskelet Disord. 2017 Nov 21;18:480. doi: 10.1186/s12891-017-1837-1 (PMC5698964; doi:10.1186/s12891-017-1837-1)

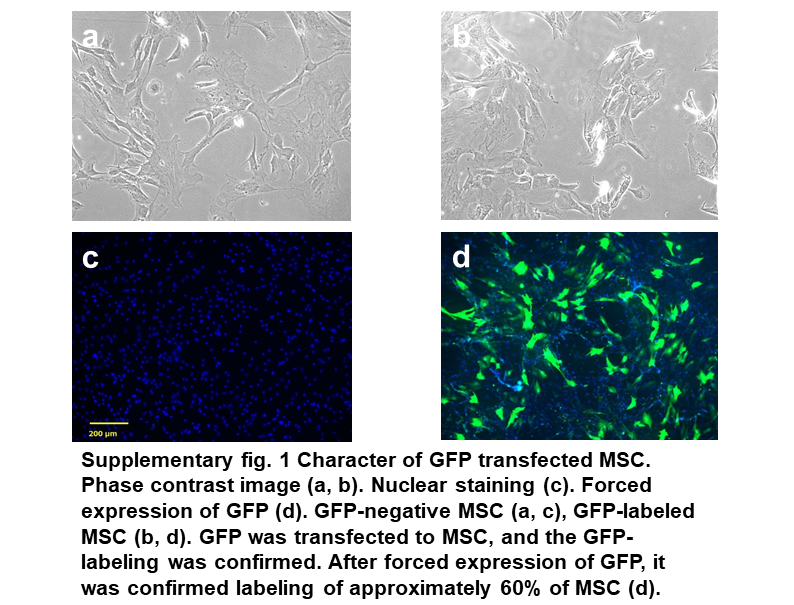

Supplement: Supplementary file 1 — Character of GFP transfected MSC. Phase contrast image (a, b). Nuclear staining (c). Forced expression of GFP (d). GFP-negative MSC (a, c), GFP-labeled MSC (b, d). GFP was transfected to MSC, and the GFP-labeling was confirmed. After forced expression of GFP, it was confirmed labeling of approximately 60% of MSC (d). (TIFF 445 kb) [file 12891_2017_1837_MOESM1_ESM.tif]

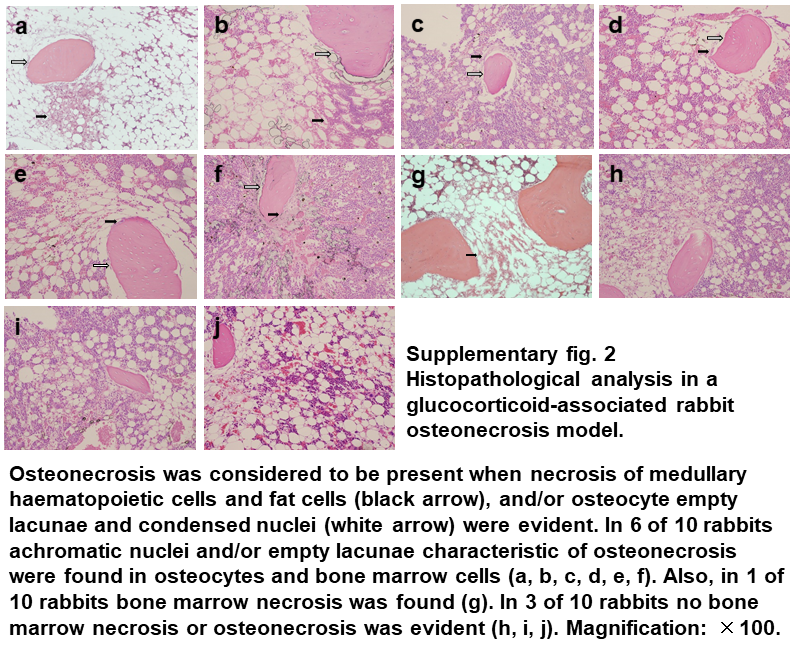

Supplement: Supplementary file 2 — Histopathological analysis in a glucocorticoid-associated rabbit osteonecrosis model. Osteonecrosis was considered to be present when necrosis of medullary haematopoietic cells and fat cells (black arrow), and/or osteocyte empty lacunae and condensed nuclei (white arrow) were evident. In 6 of 10 rabbits achromatic nuclei and/or empty lacunae characteristic of osteonecrosis were found in osteocytes and bone marrow cells (a, b, c, d, e, f). Also, in 1 of 10 rabbits bone marrow necrosis was found (g). In 3 of 10 rabbits no bone marrow necrosis or osteonecrosis was evident (h, i, j). Magnification: ×100. (TIFF 824 kb) [file 12891_2017_1837_MOESM2_ESM.tif]

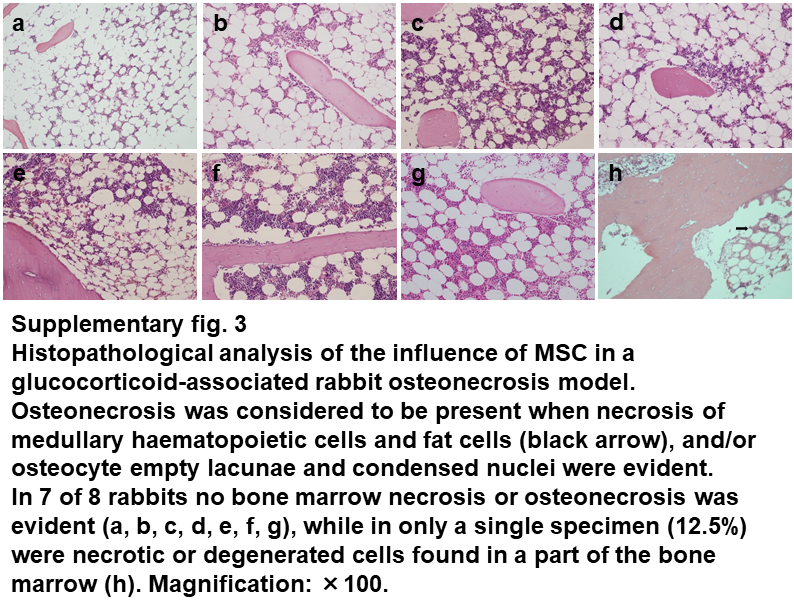

Supplement: Supplementary file 3 — Histopathological analysis of the influence of MSC in a glucocorticoid-associated rabbit osteonecrosis model. Osteonecrosis was considered to be present when necrosis of medullary haematopoietic cells and fat cells (black arrow), and/or osteocyte empty lacunae and condensed nuclei were evident. In 7 of 8 rabbits no bone marrow necrosis or osteonecrosis was evident (a, b, c, d, e, f, g), while in only a single specimen (12.5%) were necrotic or degenerated cells found in a part of the bone marrow (h). Magnification: ×100. (TIFF 650 kb) [file 12891_2017_1837_MOESM3_ESM.tif]

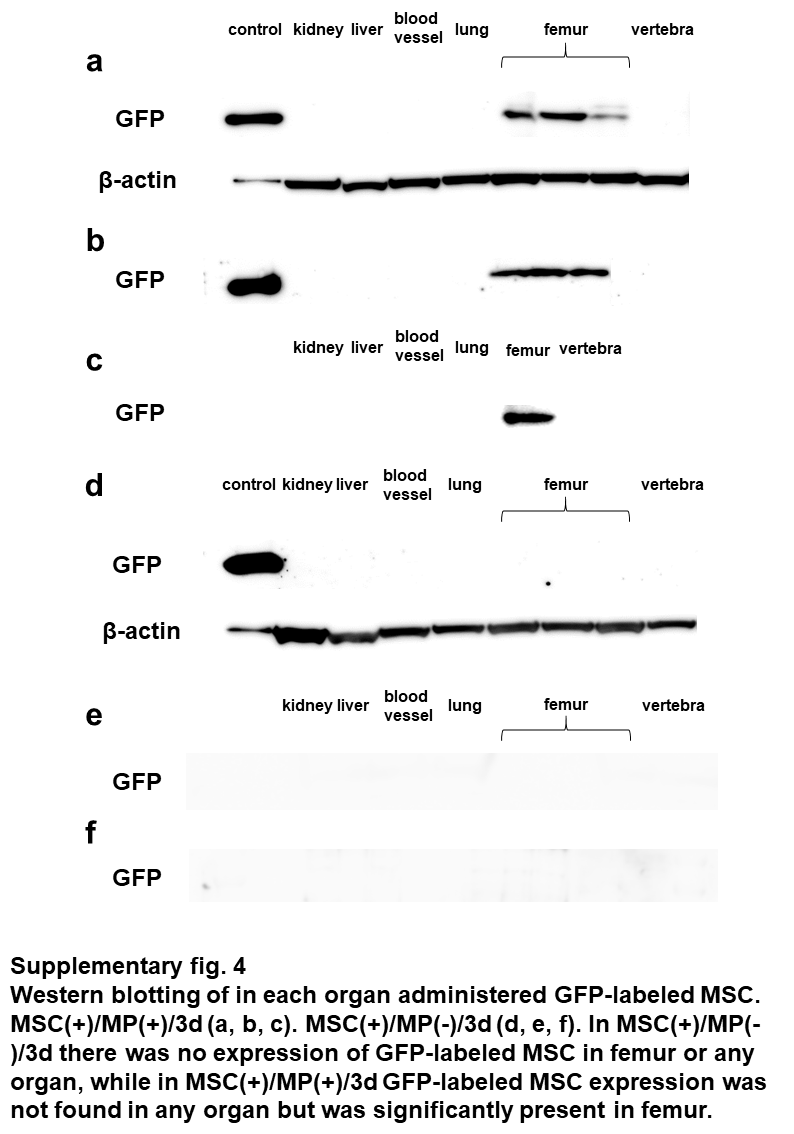

Supplement: Supplementary file 4 — Western blotting of in each organ administered GFP-labeled MSC. MSC(+)/MP(+)/3d (a, b, c). MSC(+)/MP(−)/3d (d, e, f). In MSC(+)/MP(−)/3d there was no expression of GFP-labeled MSC in femur or any organ, while in MSC(+)/MP(+)/3d GFP-labeled MSC expression was not found in any organ but was significantly present in femur. (TIFF 159 kb) [file 12891_2017_1837_MOESM4_ESM.tif]

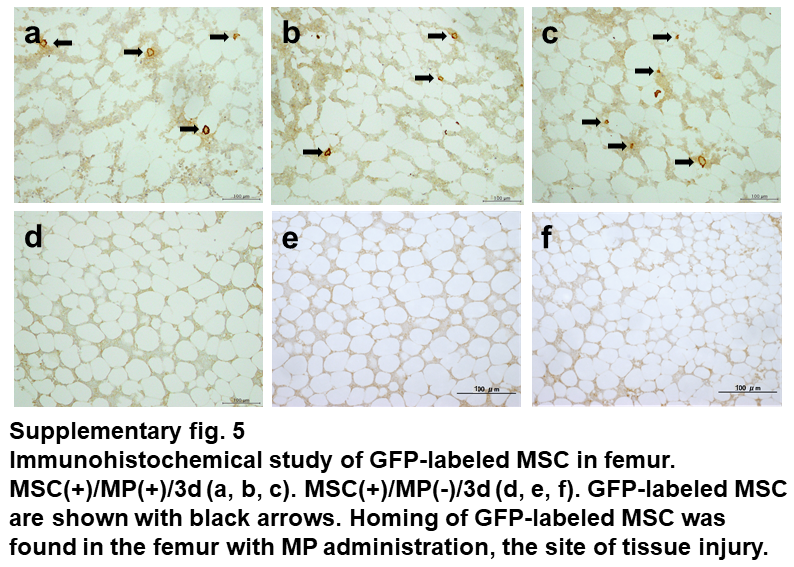

Supplement: Supplementary file 5 — Immunohistochemical study of GFP-labeled MSC in femur. MSC(+)/MP(+)/3d (a, b, c). MSC(+)/MP(−)/3d (d, e, f). GFP-labeled MSC are shown with black arrows. Homing of GFP-labeled MSC was found in the femur with MP administration, the site of tissue injury. (TIFF 677 kb) [file 12891_2017_1837_MOESM5_ESM.tif]
